# The Menace of Schistosomiasis in Nigeria: Knowledge, Attitude, and Practices Regarding Schistosomiasis among Rural Communities in Kano State

**DOI:** 10.1371/journal.pone.0143667

**Published:** 2015-11-25

**Authors:** Salwa Dawaki, Hesham M. Al-Mekhlafi, Init Ithoi, Jamaiah Ibrahim, Awatif M. Abdulsalam, Abdulhamid Ahmed, Hany Sady, Nabil A. Nasr, Wahib M. Atroosh

**Affiliations:** 1 Department of Parasitology, Faculty of Medicine, University of Malaya, Kuala Lumpur, Malaysia; 2 Azal National Research Center, Azal University for Human Development, Sana’a, Yemen; 3 Department of Parasitology, Faculty of Medicine and Health Sciences, Sana’a University, Sana’a, Yemen; 4 Department of Biology, Faculty of Natural and Applied Sciences, Umaru Musa Yar’adua University, Katsina, Katsina State, Nigeria; Tulane University, UNITED STATES

## Abstract

**Background:**

Schistosomiasis is one of the most common neglected tropical diseases, especially in the developing countries in Africa, Asia and South America, with Nigeria having the greatest number of cases of schistosomiasis worldwide. This community-based study aims to evaluate the knowledge, attitude and practices (KAP) regarding schistosomiasis among rural Hausa communities in Kano State, Nigeria.

**Methods:**

A cross-sectional study was carried out among 551 participants from Hausa communities in five local government areas in Kano State, North Central Nigeria. Demographic, socioeconomic and environmental information as well as KAP data were collected using a pre-tested questionnaire. Moreover, faecal and urine samples were collected and examined for the presence of *Schistosoma mansoni* and *S*. *haematobium* eggs respectively.

**Results:**

The overall prevalence of schistosomiasis was 17.8%, with 8.9% and 8.3% infected with *S*. *mansoni* and *S*. *haematobium* respectively, and 0.5% had co-infection of both species. Moreover, 74.5% of the participants had prior knowledge about schistosomiasis with 67.0% of them how it is transmitted and 63.8% having no idea about the preventive measures. Three-quarters of the respondents considered schistosomiasis a serious disease while their practices to prevent infections were still inadequate, with only 34.7% of them seeking treatment from clinics/hospitals. Significant associations between the KAP and age, gender, education and employment status were reported. Multiple logistic regression analysis revealed that age, gender, history of infection and educational level of the respondents were the most important factors significantly associated with the KAP on schistosomiasis among this population.

**Conclusions:**

Schistosomiasis is still prevalent among Hausa communities in Nigeria and participants’ knowledge about the disease was poor. Mass drug administration, community mobilization and health education regarding the cause, transmission and prevention of schistosomiasis and education about good personal and sanitary hygiene practices should be considered in order to significantly reduce the prevalence and morbidity of infection within these communities.

## Introduction

Schistosomiasis or bilharzias, one of the most prevalent neglected tropical diseases (NTDs), is still considered as a public health problem in many developing countries in the tropics and subtropics. About 200 million people are infected (mostly in the developing world), with about 700 million people worldwide at risk of infection [1, 2]. Over 90% of this infection occurs in sub-Saharan Africa [3]. These blood flukes are among the helminth infections that cause considerable morbidity and mortality [4]. The prevalence and intensity of schistosomiasis are highest among school-age children, adolescents and young adults [3]. Thus, the negative impacts on school performance and the debilitation caused by untreated infections demoralizes both social and economic development in endemic areas [5]. It is estimated that around 70 million disability-adjusted life years (DALYs) are forfeited annually due to schistosomiasis [6].

It is well known that lack of awareness about mode of transmission of parasitic infections increases the risk of infection. Therefore, community awareness and better understanding of the social, cultural and behavioural determinants affects the epidemiology and control of parasitic infections, and consequently aids in designing effective control strategies [7]. Moreover, participation of the targeted communities in the control efforts is one of the cardinal tools for the success and sustainability of disease control programmes [8]. Furthermore, Pablos-Mendez et al. [9] stressed the importance of researchers’ insight on subjects’ perception, which must be translated into strategic processes to move evidence-based, cost-effective interventions to reality. In low socioeconomic communities, intervention through public awareness is often recommended as a first line of action to create the enabling environment for other strategies to thrive [10].

Nigeria has the greatest number of cases of schistosomiasis worldwide, with about 29 million infected cases and about 101 million people are at risk of infection [3, 11–13]. However, there is a scarcity of research on the KAP towards schistosomiasis in the majority of the federation. Such information is crucial to identify and implement effective control measures. Within this context, the present study aims to investigate the people’s KAP regarding schistosomiasis in Kano State, North Central Nigeria.

## Materials and Methods

Ethical approval was obtained from the Medical Ethics Committee of the University of Malaya Medical Centre, Kuala Lumpur. Permission was also obtained from Kano State’s Ministry of Health, Kano State Hospitals Management Board, the respective local government authorities and the district heads of the communities. When seeking consent from the volunteers in each village, the objectives and procedures of the study were clearly explained to them in the local language, Hausa. Participants were also informed that they could withdraw from the study without any consequences. Thus, written and signed or thumb-printed informed consents were obtained from all adult participants and guardians/parents on behalf of their children before starting the survey, and these procedures were also approved by the ethics committees. All the infected individuals were treated with a single dose of 40mg/kg body weight praziquantel tablets while being observed by a researcher and a medical officer (Direct Observed Therapy).

**Fig 1:**
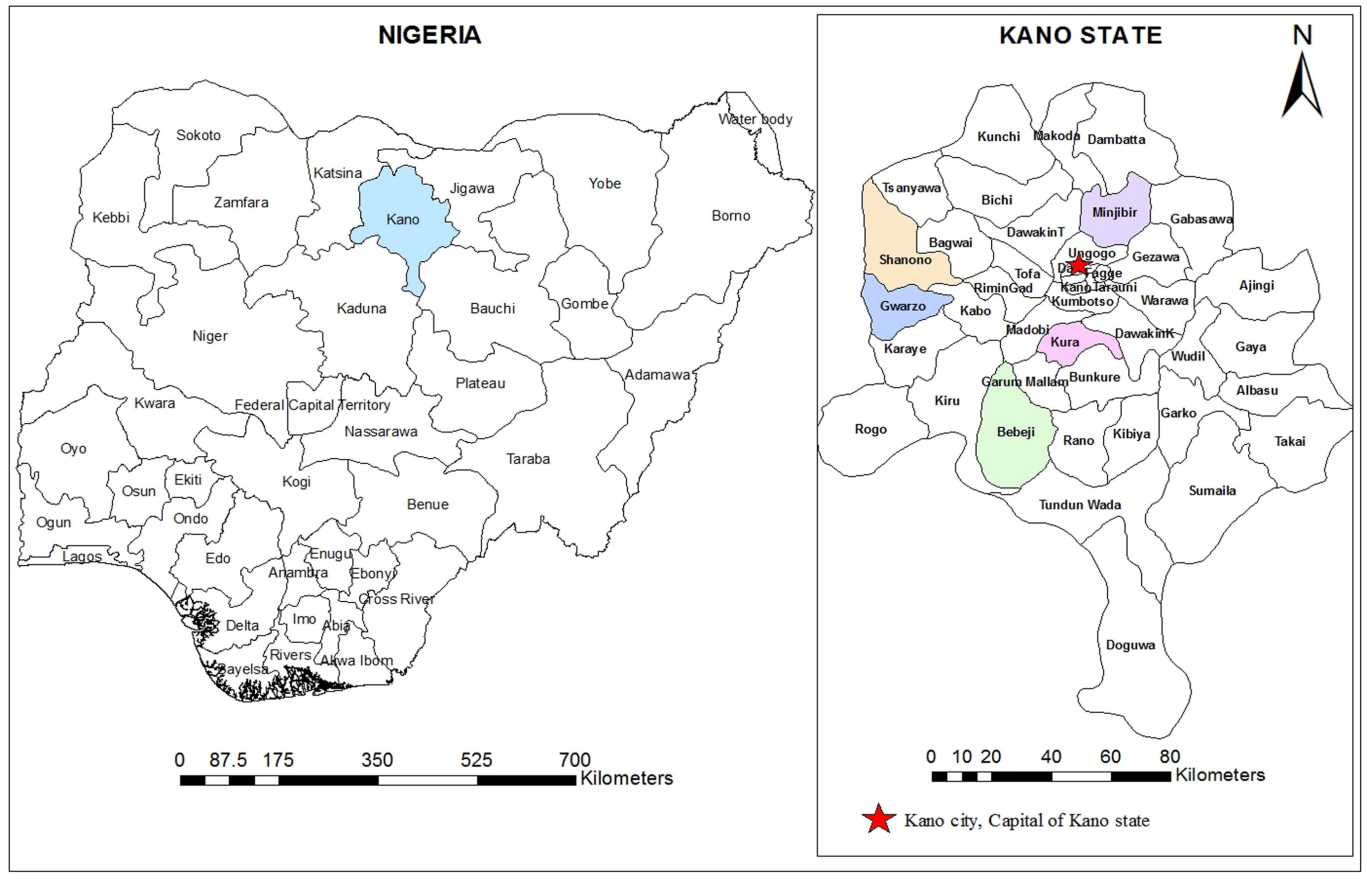
A geographic map showing the location of Kano State and the districts involved in the study. The map was created using the Esri ArcMap 10.2.1 software.

A cross-sectional community-based study was carried out between May and June 2013 among participants aged between 1 and 90 years in Kano State, North Central Nigeria. In collaboration with primary health care personnel and traditional rulers in each local government area, five districts were randomly selected from the available district list (Fig 1). Then, two villages within the selected districts were randomly considered for this study. The selected districts were Kura (8.429°E and 11.774°N), Bebeji (8.400°E and 12.366°N), Gwarzo (7.932°E and 11.915°N), Shanono (7.983°E and 12.049°N), and Minjibir (8.530°E and 12.226°N). The climate of the study area is the tropical dry-and-wet type, typical of West African savannah. The wet season lasts from May to October while the dry season extends from October to April. The annual mean rainfall ranges between 800mm and 900mm. Temperature is generally high throughout the year (maximum value reaches as high as 43°C) with the mean annual temperature at about 26°C [14].

Before the commencement of the study, the objectives and plan were explained to the heads of the selected villages in order to get their cooperation and permission to conduct the survey. Then, the heads informed all residents to gather at the area school or clinic where they received explanation about the objectives of the survey and their participation. All residents who agreed voluntarily to participate were included in this study (universal sampling). They received labeled containers and were instructed to bring their stool and urine samples the next day.

Out of 609 individuals who agreed to voluntarily participate in this study, 551 (90.5%) of individuals, aged between 1 and 90 years, successfully delivered the stool and urine samples for examinations and gave their signed written consent. Then, 505 individuals, aged ≥ 10 years, were interviewed to fill in a structured questionnaire. In these communities, children and male adolescents were seen bathing/swimming in the streams and ponds especially at midday. Although toilets were available in almost all the houses, human and animal excreta were seen around the water bodies and within farmlands.

A pre-tested questionnaire was administered to the participants in order to collect data about the demographic, socioeconomic and environmental background, personal hygiene, and history of infection. It also included questions about the KAP of the participants towards schistosomiasis. Questions on knowledge were open-ended questions, without multiple-choice answers to avoid guessing which may give a false impression of the knowledge of the participants. On the other hand, the questions on the practices were provided with multiple-choice answers to assess the frequency of doing these activities or actions. Participants were interviewed face-to-face at their home settings by two research assistants who were trained in the purpose of the study and on how to administer the questionnaire.

Stool and urine samples were collected from each participant, between 9am and 2pm, into 100 mL clean containers with wide mouth and screw-cap. On collection, the samples were properly labeled and then transported (within 5 hours of collection) in suitable cool boxes at temperature between 4 and 6°C for subsequent examination at Aminu Kano Teaching Hospital, Kano, Nigeria.

Stool samples were examined by direct microscopy, formalin ether sedimentation and Kato Katz methods for the presence of *S*. *mansoni* eggs [15]. To determine the worm burden, egg counts were taken and recorded as eggs per gram of faeces (EPG) for each positive sample and the intensity of infections was graded as heavy (≥ 400 EPG), moderate (100–399 EPG) or light (1–99 EPG) according to the criteria proposed by the WHO [16]. Similarly, urine samples were examined for haematuria using a dipstick test (Chuncheon, Korea) [17], and then centrifuged and the sediments were examined for the presence of *S*. *haematobium* eggs as described by Cheesbrough [15]. Moreover, egg counts of *S*. *haematobium* were taken and recorded as eggs per 10 millilitres urine (EP10ml), and the intensity of the infection was graded as heavy (> 50 EP10ml) or light (1–50 EP10ml) [16]. For quality control, 20% of the samples were re-examined for the presence of *Schistosoma* eggs by another parasitologist.

Data was double-entered by two different researchers into spreadsheets of IBM SPSS Statistics, version 18.0 (IBM Corporation, NY, USA). Then, a third researcher crosschecked the two data sets for accuracy and created a single data set for data analysis. Demographic, socioeconomic, environmental and behavioural characteristics were treated as categorical variables and presented as frequencies and percentages. Pearson’s Chi square test was used to examine the associations of KAP items with the demographic and socioeconomic factors. Odds ratios (OR) at 95% confidence intervals (CI) were also computed. Multivariable logistic regression analysis was used to identify key factors significantly associated with the participants’ KAP. All tests were considered significant at *P* < 0.05.

## Results

Among the 551 participants, 340 (61.7%) were males and 211 (38.3%) were females, comprising 198(35.9%) children aged below 18 years and 353 (64.1%) adults. Overall, 463 (84.0%) of the respondents had attained at least 6 years of formal education while only 270 (49.0%) were employed. Accordingly, those with an overall family monthly income of N32,000 (US$200) and above were 231(41.9%). In all the communities, houses are made mostly of mud (78%) or concrete (27%). All the houses had toilets, but most (87.3%) were traditional pit toilets, and about two-thirds of the houses had access to a piped water supply (Table 1).

**Table 1:** Demographic and socioeconomic characteristics of the participants from Kano State, Nigeria (n = 551).

Variables	N	%
Gender		
Males	340	61.7
Females	211	38.3
Age groups		
≥18 years	353	64.1
<18 years	198	35.9
Location		
Kura	127	23.0
Bebeji	119	21.6
Gwarzo	97	17.6
Shanono	99	18.0
Minjibir	109	19.8
Educational level		
Educated (formal education)	463	84.0
Non educated (no formal education)	88	16.0
Occupational status		
Working	270	49.0
Non working	281	51.0
Family monthly income		
≥ NGN32000	231	41.9
< NGN32000 (low)	320	58.1
Family size		
≤ 10 members	267	48.5
> 10 members	284	51.5
Toilet facility		
Pour flush system	70	12.7
Pit (ground dug)	481	87.3
Drinking water		
Safe (treated)	356	64.6
Unsafe	195	35.4
Have contact with a water body	257	50.9
Had history of infection	214	38.8
Experienced haematuria	219	39.7
Experienced blood in stool	217	39.4

NGN, Nigerian Naira; (US$1 = NGN 165).

Overall, 17.8% (98/551) of the participants were found to be positive for schistosomiasis. Of them, 49 (8.9%) were infected with *S*. *mansoni* and 46 (8.3%) were infected with *S*. *haematobium;* 3 (0.5%) had co-infection of both species. Of the 49 *S*. *haematobium*-positive samples, 15 (30.6%) were of heavy intensity with a mean EP10ml of 98 eggs while 34 (69.4%) cases were of light intensity with a mean EP10ml of 32 eggs. Likewise, 8 (15.4%) and 2 (3.8%) *S*. *mansoni* cases were of moderate and heavy intensity with a mean of EPG of 128 and 458 eggs, respectively. Moreover, 42 (80.8%) *S*. *mansoni* cases were light infections with a mean EPG of 65 eggs. The infection was significantly higher among males than females (20.6% vs 13.3%; *P* = 0.029). Moreover, the highest prevalence was reported among participants aged 10–18 years (27.8%) while those aged 31–50 years had the lowest prevalence (10.6%).

This study revealed that there was a considerable awareness of schistosomiasis (74.5%, 376/505), and people get to hear about the disease mostly through family or neighbours (71.3%) followed by mass media (21.5%). However, understanding the source of infection, transmission and preventive measures was comparably not much. For instance, only 44.7% (168/376) of the participants knew that worms cause the disease and related the infection to water body sources. Moreover, a considerable portion of the subjects (59.0%) recognized haematuria as a symptom but very few mentioned blood in stools and other symptoms. Likewise, about one-third (38.6%) of them did not know any of the symptoms and 67.0% had no idea how it is transmitted with an overwhelming 63.8% (240/376) ignorant on how to avoid becoming infected.

Table 2 shows that respondents aged < 18 years had better knowledge about the cause of schistosomiasis. The percentage of respondents aged < 18 years who mentioned worms (54.7% vs 40.7%; *P* = 0.014) and polluted water (58.5% vs 46.7%; *P* = 0.039) was significantly higher than in those aged ≥ 18 years. Similarly, it was found that male respondents had significantly higher knowledge about schistosomiasis than females (81.3% vs 63.6%; *P* < 0.001). Moreover, percentages of males who mentioned haematuria as a sign of schistosomiasis (63.9% vs 49.2%; *P* = 0.006), contaminated water as a mode of transmission (31.7% vs 20.2%; *P* = 0.019), and medication as a curative measure (34.5% vs 12.1%; *P* < 0.001) were significantly higher when compared to female respondents.

**Table 2:** Association of knowledge of the participants about schistosomiasis with their age and gender (n = 376).

Variables	Age (years)				Gender			
	≥ 18	< 18	OR	95% CI	Female	Male	OR	95% CI
Heard about schistosomiasis	270 (76.5)	106 (69.7)	0.71	0.46, 1.08	124 (63.6)	252 (81.3)	2.49	1.65, 3.74*
**Causes**								
Worms	110 (40.7)	58 (54.7)	1.76	1.12, 2.77*	38 (30.6)	130 (51.6)	2.41	1.53, 3.80*
Polluted water	126 (46.7)	62 (58.5)	1.61	1.02, 2.54*	43 (34.7)	145 (57.5)	2.55	1.63, 4.00*
Salty or sour food	53 (19.6)	18 (17.0)	0.84	0.47, 1.51	19 (15.3)	52 (20.6)	1.43	0.81, 2.56
Poor personal hygiene	20 (7.4)	0 (0.0)	NA	NA	7 (5.6)	13 (5.2)	0.91	0.35, 2.34
Do not know	67 (24.8)	5 (4.7)	0.15	0.07, 0.38*	38 (30.6)	34 (13.5)	0.35	0.21, 0.30*
**Signs and symptoms**								
Haematuria	157 (58.1)	65 (61.3)	1.14	0.72, 1.81	61 (49.2)	161 (63.9)	1.83	1.18, 2.83*
Blood in stool	41 (15.2)	15 (14.3)	0.92	0.49, 1.75	14 (11.3)	42 (16.7)	1.57	0.82, 3.00
Burning urination	14 (5.2)	1 (0.9)	0.21	0.04, 1.60	2 (1.6)	13 (5.2)	3.32	0.74, 14.93
Abdominal pain	12 (4.4)	1 (0.9)	0.17	0.03, 1.34	2 (1.6)	11 (4.4)	2.78	0.61, 12.76
Do not know	108 (40.0)	37 (34.9)	0.80	0.51, 1.28	62 (50.0)	83 (32.9)	0.49	0.32, 0.76*
**Transmission**								
Contaminated water	77 (28.5)	28 (26.4)	0.90	0.54, 1.49	25 (20.2)	80 (31.7)	1.84	1.10, 3.08*
Sharing toilet	19 (7.0)	4 (3.8)	0.52	0.17, 1.56	5 (4.0)	18 (7.1)	1.83	0.66, 5.05
Poor personal hygiene	8 (3.0)	1 (0.9)	0.31	0.05, 2.52	1 (0.8)	8 (3.2)	4.03	0.50, 32.51
Do not know	178 (65.9)	74 (69.8)	1.20	0.74, 1.94	90 (72.6)	162 (64.3)	0.68	0.43, 1.09
**Prevention**								
Medication	64 (23.7)	38 (35.8)	1.80	1.11, 2.93*	15 (12.1)	87 (34.5)	3.83	2.11, 6.97*
Avoid contact with contaminated water	20 (7.4)	3 (2.8)	0.37	0.11, 1.25	4 (3.2)	19 (7.5)	2.45	0.81, 7.35
Good personal hygiene	12 (4.4)	1 (0.09)	0.21	0.04, 1.59	3 (2.4)	10 (4.0)	1.67	0.45, 6.17
Do not know	184 (68.1)	56 (52.8)	0.52	0.33, 0.83*	98 (79.0)	142 (56.3)	0.34	0.21, 0.56*

All values are number (%). OR, Odds ratio. CI, Confidence interval. NA, Not applicable.

* Significant association (*P* < 0.05).

Table 3 shows that the proportion of people who mentioned haematuria as a symptom of the infection was significantly higher among the educated respondents compared to those without formal education (62.4% vs 40.4%; *P* = 0.002). Furthermore, individuals with formal education were twice as likely than their counterparts to consider contaminated water as the principal means of schistosomiasis transmission (30.1% vs 15.8%; *P* = 0.027) and deworming as a preventive measure (29.2% vs 15.8%; *P* = 0.037). Grouping based on employment status, the subject’s knowledge of avoiding contaminated water as prevention was significantly higher among the employed respondents (8.7% vs 3.3%; *P* = 0.029).

**Table 3:** Association of knowledge of the participants about schistosomiasis with their education and employment status (n = 376).

Variables	Educational level				Employment status			
	Non educated	Educated	OR	95% CI	Not working	Working	OR	95% CI
Heard about schistosomiasis	57 (80.3)	319 (73.5)	0.68	0.37, 1.27	181 (73.3)	195 (75.6)	1.13	0.76, 1.68
**Causes**								
Worms	20 (35.1)	148 (46.4)	1.60	0.89, 2.88	86 (47.5)	82 (42.1)	0.80	0.53, 1.21
Polluted water	24 (42.1)	164 (51.4)	1.46	0.82, 2.57	95 (52.5)	93 (47.7)	0.83	0.55, 1.24
Salty or sour food	7 (12.3)	64 (20.1)	1.79	0.78, 4.14	32 (17.7)	39 (20.0)	1.16	0.70, 1.96
Poor personal hygiene	4 (7.0)	16 (5.0)	0.70	0.23, 2.17	9 (5.0)	11 (5.6)	1.14	0.46, 2.83
Do not know	16 (28.1)	56 (17.6)	0.55	0.29, 1.04	26 (14.4)	46 (23.6)	1.84	1.08, 3.13*
**Signs and symptoms**								
Haematuria	23 (40.4)	199 (62.4)	2.45	1.38, 4.36*	108 (59.7)	114 (58.5)	0.95	0.63, 1.44
Blood in stool	8 (14.0)	48 (15.0)	1.09	0.48, 2.43	24 (13.3)	32 (16.4)	1.28	0.72, 2.28
Burning urination	1 (1.8)	14 (4.4)	2.57	0.33, 19.94	7 (3.9)	8 (4.1)	1.06	0.38, 3.00
Abdominal pain	1 (1.8)	12 (3.8)	2.19	0.28, 17.17	7 (3.9)	6 (3.1)	0.79	0.26, 2.39
Do not know	33 (57.9)	112 (35.1)	0.39	0.22, 0.70*	68 (37.6)	77 (39.5)	1.08	0.72, 1.64
**Transmission**								
Contaminated water	9 (15.8)	96 (30.1)	2.30	1.08, 4.87*	46 (25.4)	59 (30.3)	1.27	0.81, 2.00
Sharing toilet	5 (8.8)	18 (5.6)	0.62	0.22, 1.75	12 (6.6)	11 (5.6)	0.84	0.36, 1.96
Poor personal hygiene	1 (1.8)	8 (2.5)	1.44	0.18, 11.74	4 (2.2)	5 (2.6)	1.16	0.31, 4.41
Do not know	42 (73.7)	210 (65.8)	0.69	0.36, 1.30	127 (70.2)	125 (64.1)	0.76	0.49, 1.17
**Prevention**								
Medication	9 (15.8)	93 (29.2)	2.20	1.04, 4.65*	53 (29.3)	49 (25.1)	0.81	0.51, 1.28
Avoid contact with contaminated water	4 (7.0)	19 (6.0)	0.84	0.28, 2.56	6 (3.3)	17 (8.7)	2.79	1.07, 7.23*
Good personal hygiene	3 (5.3)	10 (3.1)	0.58	0.16, 2.19	3 (1.7)	10 (5.1)	3.21	0.87, 11.85
Do not know	42 (73.7)	198 (62.1)	0.58	0.31, 1.01	108 (59.7)	132 (67.7)	1.42	0.93, 2.16

All values are number (%). OR, Odds ratio. CI, Confidence interval.

* Significant association (*P* < 0.05)

Generally, the detrimental nature of schistosomiasis was recognized by 75.0% (282/376) of the respondents who agreed that schistosomiasis is a serious disease. With regards to practices, 50.9% (257/505) of the respondents admitted to having had contact with a water body, for domestic purposes (68.1%) and swimming (25.7%) as the most frequently reported reasons. Moreover, about two-thirds (68.9%) of the participants revealed that they live near a water body (< 250 meter), 34.9% use unsafe drinking water, and 36.6% use unsafe water for domestic purposes. Inclination towards self-medication among the respondents was substantial (47.7%) followed by seeking treatment from hospitals (34.7%) and using traditional medicine (12.7%). Yet 5.0% indicated that they do nothing.

Table 4 shows that 67.9% of the respondents aged below 18 years considered the disease serious compared to 77.8% of those aged ≥ 18 years (*P* = 0.047). Likewise, respondents aged below 18 years had significantly higher contact with a water body (mainly for swimming) compared to those aged ≥ 18 years (65.8% vs 44.5%; *P* < 0.001). Interestingly, self-medication for such symptoms was significantly higher among those aged below 18 years compared to their older counterparts (57.9% vs 43.3%; *P* = 0.003).

**Table 4:** Association of attitude and practices of the participants towards schistosomiasis with their age and gender.

Variables	Age (years)				Gender			
	≥ 18	< 18	OR	95% CI	Female	Male	OR	95% CI
**Attitude (n = 376)**								
Schistosomiasis is a serious disease	210 (77.8)	72 (67.9)	0.61	0.37, 0.98*	83 (66.9)	199 (79.0)	1.86	1.15, 3.00*
**Practices (n = 505)**								
Using unsafe drinking water	118 (33.4)	58 (38.2)	1.23	0.83, 1.82	63 (32.3)	113 (36.5)	1.20	0.82, 1.76
Using unsafe water for domestic purposes	128 (36.3)	57 (37.5)	1.06	0.71, 1.56	67 (34.4)	118 (38.1)	1.17	0.81, 1.71
Living near water body	252 (71.4)	96 (63.2)	0.69	0.46, 1.03	133 (68.2)	215 (69.4)	1.06	0.71, 1.55
Had contact with a water body	157 (44.5)	100 (65.8)	2.40	1.62, 3.57*	85 (43.6)	172 (55.5)	1.61	1.12, 2.31*
Reasons for contact								
Swimming	13 (3.7)	53 (34.9)	14.00	7.33, 26.72*	4 (2.1)	62 (20.0)	11.94	4.27, 33.39*
Fishing	11 (3.1)	2 (1.3)	0.42	0.10, 1.89	1 (0.5)	12 (3.9)	7.81	1.01, 42.56*
Domestic purposes	131 (37.1)	44 (28.9)	0.69	0.46, 1.04	79 (40.5)	96 (31.0)	0.66	0.45, 1.01
Treatment-seeking behavior								
Hospital/clinic	133 (37.7)	42 (27.6)	0.63	0.42, 0.96*	56 (28.7)	119 (38.4)	1.55	1.05, 2.27*
Traditional medicine	47 (13.3)	17 (11.2)	0.82	0.45, 1.48	21 (10.8)	43 (13.9)	1.33	0.77, 2.33
Self-medication	153 (43.3)	88 (57.9)	1.80	1.22, 2.64*	102 (52.3)	139 (44.8)	0.74	0.52, 1.06
Do nothing	20 (5.7)	5 (3.3)	0.57	0.21, 1.54	16 (8.2)	9 (2.9)	0.34	0.15, 0.77*

All values are number (%). OR, Odds ratio. CI, Confidence interval.

* Significant association (*P* < 0.05).

According to gender, considerably more male respondents agreed to the seriousness of the disease compared to female respondents (79.0% vs 66.9%; *P* = 0.011). Likewise, the percentage of male respondents who had contact with a water body (swimming) was significantly higher than their female peers (55.5% vs 43.6%; *P* = 0.009). About one-third (38.4%) of the males declared going to hospital when suffering abdominal pain and/or haematuria compared to 28.7% of the females (*P* = 0.024). In the same vein, the percentage of those who do nothing for such signs and symptoms was significantly lower among male compared to female respondents (2.9% vs 8.2; *P* = 0.007).

Table 5 shows that the awareness of the seriousness of schistosomiasis was significantly higher among the educated and employed respondents compared to their non-educated and unemployed peers (OR = 2.59; 95% CI = 1.44, 4.66 and OR = 1.72; 95% CI = 1.09, 2.23, respectively). Moreover, a significantly lower percentage of employed respondents had indicated that they had contact with a water body compared to the unemployed respondents (OR = 0.54; 95%CI = 0.38, 0.77). By contrast, a significantly higher percentage of educated respondents had a swimming history in these water bodies (OR = 5.97; 95% CI = 1.43, 24.95). With regards to treatment-seeking behaviour, the percentage of respondents who do nothing to treat urinary and intestinal signs and symptoms of schistosomiasis was significantly lower among educated respondents compared to their non-educated counterparts (OR = 0.40; 95%CI = 0.16, 0.99).

**Table 5:** Association of attitude and practices of the participants towards schistosomiasis with their education and employment status.

Variables	Educational level				Employment status			
	Non educated	Educated	OR	95% CI	Not working	Working	OR	95% CI
**Attitude (n = 376)**								
Schistosomiasis is a serious disease	33 (57.9)	249 (78.1)	2.59	1.44, 4.66*	123 (68.0)	159 (81.5)	1.72	1.09, 2.23*
**Practices (n = 505)**								
Using unsafe drinking water	21 (29.6)	155 (35.7)	1.32	0.77, 2.28	87 (35.2)	89 (34.5)	0.97	0.67, 1.40
Using unsafe water for domestic purposes	27 (38.0)	158 (36.4)	0.93	0.56, 1.57	91 (36.8)	94 (36.4)	0.98	0.68, 1.41
Living near water body	46 (64.8)	302 (69.6)	1.24	0.73, 2.11	161 (65.2)	187 (72.5)	1.41	0.96, 2.05
Had contact with a water body	37 (52.1)	220 (50.7)	0.95	0.56, 1.56	145 (58.7)	112 (43.4)	0.54	0.38, 0.77*
Reasons for contact								
Swimming	2 (2.8)	64 (14.7)	5.97	1.43, 24.95*	57 (23.1)	9 (3.5)	0.12	0.07, 0.25*
Fishing	4 (5.6)	9 (2.1)	0.36	0.11, 1.18	7 (2.8)	6 (2.3)	0.82	0.27, 2.46
Domestic purposes	31 (43.7)	144 (33.2)	0.64	0.39, 1.07	80 (32.4)	95 (36.8)	1.22	0.84, 1.76
Treatment-seeking behaviour								
Hospital/clinic	21 (29.6)	154 (35.5)	1.31	0.76, 2.26	79 (32.0)	96 (37.2)	1.26	0.87, 1.82
Traditional medicine	9 (12.7)	55 (12.7)	1.00	0.47, 2.13	33 (13.4)	31 (12.0)	0.89	0.52, 1.50
Self-medication	34 (47.9)	207 (47.7)	0.99	0.60, 1.64	122 (49.4)	119 (46.1)	0.88	0.62, 1.24
Do nothing	7 (9.9)	18 (4.1)	0.40	0.16, 0.99*	13 (5.3)	12 (4.7)	0.88	0.39, 1.96

All values are number (%). OR, Odds ratio. CI, Confidence interval.

* Significant association (*P* < 0.05).

The results of multiple logistic regression analyses for the factors significantly associated with the KAP on schistosomiasis among these participants showed that age, gender, educational level and the history of infection were the key factors significantly associated with KAP among the respondents. The results indicated that male respondents had significantly higher odds of hearing about schistosomiasis (OR = 2.32; 95% CI = 1.28, 4.21) and had significantly higher knowledge about haematuria (OR = 1.49; 95%CI = 1.26, 3.91) as a symptom of schistosomiasis when compared with non-educated respondents. Likewise, respondents who had a history of schistosomiasis showed higher odds of having knowledge about haematuria (OR = 1.87; 95% CI = 1.19, 2.93) and swimming or playing in open water sources as a mode of transmission (OR = 2.07; 95% CI = 1.12, 5.13) when compared with those with no previous history of infection. Moreover, respondents aged below 18 years were less likely to hear about schistosomiasis (OR = 0.41; 95% CI = 0.18, 0.91).

## Discussion

The present study revealed that the prevalence rate of schistosomiasis in the study area was 17.8% with no significant difference in the prevalence of urogenital (8.3%) and intestinal schistosomiasis (8.9%). This prevalence is in accordance with prevalence rates reported by previous studies; 11.5% in Adamawa State [18], 15.3% in Ebonyi State [19], 17.4% in Oyo State [20], and 18.7% in Plateau and Nasarawa states of Nigeria [21]. However, higher prevalence rates were reported earlier in the same state, Kano [22–24].

With regards to the KAP towards schistosomiasis, our findings showed that the respondents were conversant with schistosomiasis, especially urinary schistosomiasis; about three-quarters of the respondents have prior knowledge of schistosomiasis. This could be attributed to the fact that schistosomiasis is endemic in Nigeria; the high percentage of self-reported history of infection among the participants supports the endemicity of infection in these communities. In accordance, few previous reports elsewhere showed variation in the level of awareness amongst the Nigerian population; 33.8%-42.0% in Delta State, Southeastern Nigeria [25, 26], and 64.4% in Ogun and Niger states along the middle belt and southwestern region [27]. In comparison with findings from other countries, poor knowledge of schistosomiasis and its causes was reported in Malawi and Kenya [28, 29] while a high level of awareness (80%) was reported in Zimbabwe [30].

The present study revealed that the majority of the respondents did not know the causes, mode of transmission, signs and symptoms, and preventive measures of schistosomiasis. This indicates lack of health education among the targeted populations which should be provided during mass drug administration campaigns. Even though half of those respondents recognized polluted water bodies as infection foci, only 6.1% (23/376) consider avoiding contaminated water as a preventive measure. A previous study in Anambra State observed that most of the subjects understand that water bodies transmit the infection and they desist from drinking from it; however, they still carry out other domestic activities in that water [31]. Correspondingly, previous studies in other endemic areas of Africa, Yemen and Brazil indicated similar observations [32–35]. This may indicate that awareness alone does not necessarily result in behavioural changes, which are often more difficult to be achieved, requiring long periods of time to ensure compliance with healthier practices [36].

With regards to knowledge about signs and symptoms, our findings showed that 59% of the respondents mentioned haematuria while about one-third of them could not associate the infection with any symptom. It is also worth noting that respondents’ knowledge about the symptoms of intestinal schistosomiasis was negligible as only 14.9% mentioned blood in stools. This is in agreement with previous studies in Yemen, Egypt, Western Cote d’Ivoire and Senegal [34, 37–39]. This could be due to the disease being frequently confused with other diseases exhibiting similar symptoms. The local name for schistosomiasis in Hausa language, “*Tsargiya*”, is synonymous with urinary schistosomiasis, meaning blood in urine, and this may also explain the better knowledge about haematuria. Interestingly, we found that only 27.9% of the studied respondents associated the disease with contact with contaminated water while none of them indicated the role of snails in the transmission of schistosomiasis. A previous study in Ogun and Niger states found that none of the respondents mentioned avoiding urination/defaecation in water bodies as a preventive measure [27].

Our findings showed that majority of the respondents knew about schistosomiasis from their family members or neighbours. This may explain the misconceptions and diverging views about causation, transmission and symptomatology of the disease among these people. For instance, 18.9% of the respondents believe that the infection is caused by eating salty or sour food or that it can be spread by sharing a toilet with an infected person. Moreover, a community leader believed that it is God’s wish, suggesting that one cannot prevent it. Indicating haematuria, an elderly lady said: ‘It is just something that children, especially boys, do but will outgrow later’. A previous study among grade three primary school children in Zimbabwe reported some misconceptions of the causes of schistosomiasis, such as eating too much salty food, stepping in a witch’s place, jumping over fire or eating green mangoes. It concluded that parents may inform their children of these misconceptions to prevent them from the real dangers associated with fire or from eating fruits before they are ripe [40]. Likewise, a previous study among 207 household heads in Cote d’Ivoire found that a large proportion of them believed that avoiding unripe fruit consumption is a preventive measure of intestinal schistosomiasis [38]. Interestingly, a study in Delta State, Southern Nigeria reported that some respondents mentioned maturity and witchcraft as causes, and sexual contact with an infected person as modes of transmission of schistosomiasis [41].

The present study showed that knowledge about schistosomiasis was significantly higher among males, those aged below 18 years, and educated and working respondents compared to their counterparts. Despite high prevalence of schistosomiasis, a previous study in Western Kenya found that most respondents stated having heard about schistosomiasis but very few had knowledge of signs and symptoms, causes, transmission and prevention of infection [27]. These findings are in agreement with a previous study from China and Uganda that sex, age, educational level, occupation and income were significant predictors of knowledge of schistosomiasis [41, 42]. Our survey on the attitude towards the disease revealed that three-quarters of the respondents agreed that schistosomiasis is a serious disease. As expressed by one respondent, ‘Yes, it is a serious disease. From experience, I know it is very painful and who knows how much blood one loses daily?’ Besides the pain experienced during urination and defaecation, high perception of the devastating nature of this disease was probably due to the fact that it is customary for people in these communities to associate anything that results in blood coming out of the body as very serious.

Association of water contact with risk and transmission of schistosomiasis is well documented [1, 43–45]. However, a previous study reported that age and sex were the only two significant factors that help in predicting the level of *S*. *haematobium* infection while an individual's water contact pattern was not significant [46]. In the present study, half of the respondents claimed previous contact with water bodies, mainly for swimming and domestic purposes. Among boys, we observed that group swimming was an important recreational activity, especially at midday. An earlier study in Northern Nigeria revealed that males under the age of 21 years were responsible for more than 77% of water contamination by *Schistosoma* eggs and suggested that serious attention on group swimming by young males should be considered by any control strategy [47]. Although all the respondents admitted to having toilet facilities at their households (either pit or pour flush), they still practice open or indiscriminate urination/defaecation along the watersides and in nearby bushes. A teenager argued that ‘it is not feasible to go back home just to answer nature’s call’ while another claimed ‘Why burden ourselves when there is water around to clean?’ Similar findings were reported by previous studies in Nigeria [31, 27]. Based on these findings, it can be deduced that provision of toilets alone would not prevent this undisciplined manner and public enlightenment on the importance of using the toilets in controlling schistosomiasis and other parasitic infections should be provided to the targeted population.

Despite the reported high attitude of the seriousness of infection among the studied population, the tendency to seek medication from a hospital/clinic is not substantial as only 34.7% of the respondents seek treatment for haematuria at the nearest hospital/clinic. Our findings showed that the majority of the respondents prefer self-medication. In this context, self-medication means buying drugs from shops (called chemists) or from drug hawkers on the streets. A similar habit was observed among residents of Ogun and Niger states who testified to going to hospital only after the failure of other options [27]. Some of the respondents claimed going to the ‘chemist’ is cheaper, easier and they feel more comfortable explaining their health problems to the more familiar practitioners. A previous study among schoolchildren in Southwestern Nigeria showed that over 80% of the children claimed urinary schistosomiasis is a serious disease, yet did not complement their knowledge with appropriate treatment-seeking behaviour [48]. Perhaps the inclination towards self-medication or traditional medicine is sometimes due to poverty or inaccessibility of functioning hospitals/clinics.

Further investigation into the association of treatment-seeking behaviour with the respondents’ characteristics revealed that seeking treatment from hospitals/clinics was significantly higher among males and those aged ≥ 18 years compared to females and those aged below 18 years. In Hausa rural communities, females are prohibited from going outside without permission of the head of household (husband/father), even to hospitals. Moreover, women are less self-sufficient, thus they depend on the males for financial support to seek treatment. As a limitation, the present study had to rely on a single faecal sample instead of the ideal three consecutive samples. Thus, the prevalence rates of intestinal schistosomiasis as well as the co-infection with both species are likely to be underestimated due to the temporal variation in egg excretion over hours and days. Rural communities in Nigeria share similar socioeconomic and health profiles, thus we believe that our findings are generalisable to the entire rural population in Nigeria. However, further studies in other States are required to confirm this conjecture.

## Conclusions

The present study shows that schistosomiasis is still prevalent among Hausa communities in Kano State, Nigeria. The reported prevalence (17.8%) was low when compared to previous studies and this may indicate the success of the National Schistosomiasis Control Programme. However, our findings show that respondents’ knowledge about the cause, transmission, symptoms and prevention of schistosomiasis was inadequate and this could be a challenging obstacle to the elimination of schistosomiasis from these communities. Besides mass drug administration, school and community-based health education regarding good personal hygiene and good sanitary practices is imperative among these communities in order to significantly reduce the transmission and morbidity of schistosomiasis.
